# Mannan-MOG35-55 Reverses Experimental Autoimmune Encephalomyelitis, Inducing a Peripheral Type 2 Myeloid Response, Reducing CNS Inflammation, and Preserving Axons in Spinal Cord Lesions 

**DOI:** 10.3389/fimmu.2020.575451

**Published:** 2020-11-19

**Authors:** Anastasia Dagkonaki, Maria Avloniti, Maria Evangelidou, Irini Papazian, Ioannis Kanistras, Vivian Tseveleki, Fotis Lampros, Theodore Tselios, Lise Torp Jensen, Wiebke Möbius, Torben Ruhwedel, Maria-Eleni Androutsou, John Matsoukas, Maria Anagnostouli, Hans Lassmann, Lesley Probert

**Affiliations:** ^1^ Laboratory of Molecular Genetics, Department of Immunology, Hellenic Pasteur Institute, Athens, Greece; ^2^ Department of Chemistry, University of Patras, Patras, Greece; ^3^ Department of Clinical Medicine, Aarhus University, Aarhus, Denmark; ^4^ Electron Microscopy Core Unit, Department of Neurogenetics, Max Planck Institute of Experimental Medicine, Göttingen, Germany; ^5^ Research and Development Department, Vianex S.A., Nea Erythrea, Greece; ^6^ Immunogenetics Laboratory, First Department of Neurology, Aeginition Hospital, National and Kapodistrian University of Athens, Athens, Greece; ^7^ Department of Neuroimmunology, Center for Brain Research, Medical University of Vienna, Vienna, Austria

**Keywords:** MOGAD, multiple sclerosis, M2 macrophages, humanized DR2 mice, neuroprotection, Ym1/Chi3l3, antigen-specific immunotherapy, PD-L1

## Abstract

CNS autoantigens conjugated to oxidized mannan (OM) induce antigen-specific T cell tolerance and protect mice against autoimmune encephalomyelitis (EAE). To investigate whether OM-peptides treat EAE initiated by human MHC class II molecules, we administered OM-conjugated murine myelin oligodendrocyte glycoprotein peptide 35-55 (OM-MOG) to humanized HLA-DR2b transgenic mice (DR2b.Ab°), which are susceptible to MOG-EAE. OM-MOG protected DR2b.Ab° mice against MOG-EAE by both prophylactic and therapeutic applications. OM-MOG reversed clinical symptoms, reduced spinal cord inflammation, demyelination, and neuronal damage in DR2b.Ab° mice, while preserving axons within lesions and inducing the expression of genes associated with myelin (*Mbp*) and neuron (*Snap25*) recovery in B6 mice. OM-MOG-induced tolerance was peptide-specific, not affecting PLP178-191-induced EAE or polyclonal T cell proliferation responses. OM-MOG-induced immune tolerance involved rapid induction of PD-L1- and IL-10-producing myeloid cells, increased expression of *Chi3l3* (Ym1) in secondary lymphoid organs and characteristics of anergy in MOG-specific CD4^+^ T cells. The results show that OM-MOG treats MOG-EAE in a peptide-specific manner, across mouse/human MHC class II barriers, through induction of a peripheral type 2 myeloid cell response and T cell anergy, and suggest that OM-peptides might be useful for suppressing antigen-specific CD4^+^ T cell responses in the context of human autoimmune CNS demyelination.

## Highlights

- Mannan-MOG35-55 treats MOG-EAE in DR2b.Ab° mice
- Mannan-MOG35-55 treatment reverses CNS inflammation and preserves axons
- Mannan-MOG35-55 induces peripheral M2 myeloid cells and T cell anergy
- Myelin-specific T cells in blood of Greek MS patients

## Introduction

Autoimmune demyelinating disorders of the central nervous system (CNS) are a major cause of chronic neurologic disability in young adults. They include multiple sclerosis (MS), for which the autoimmune targets of CNS-infiltrating T and B lymphocytes are not yet fully understood (1), myelin oligodendrocyte glycoprotein (MOG) antibody associated disorders (MOGAD) which were previously considered to be part of the MS spectrum and in which the oligodendrocyte protein MOG is a major candidate autoantigen (2, 3), and neuromyelitis optica spectrum disorders (NMOSD) with an autoimmune response that targets aquaporin-4 on astrocytes. MS and MOGAD share many features including inflammatory demyelination in brain, oligodendrocyte death and infiltration by T and B lymphocytes and macrophages, and are distinguishable mainly by clinical, pathological and radiological criteria, and the presence of serum MOG IgG antibodies and predominance of brain-infiltrating CD4^+^ over CD8^+^ T cells in MOGAD (3, 4). Experimental autoimmune encephalomyelitis (EAE) models show many of the immune and pathological features of MOGAD and possibly also of MS (3, 5, 6). CD4^+^IFN-γ- (Th1) and CD4^+^IL-17-producing (Th17) specific for MOG35-55 peptide are sufficient to induce inflammatory demyelination in EAE (7), while MOG-specific antibodies further enhance demyelination in the context of myelin-specific CD4^+^ T cell inflammation (8). Therapeutic strategies that aim to re-establish peripheral tolerance selectively in myelin specific CD4^+^ T cells are therefore a promising way forward for therapy in human autoimmune demyelination.

Early studies to induce peripheral T cell tolerance in MS focused on controlling myelin-specific T cells by directly targeting the T cell receptor (TCR). Delivery of myelin protein or peptide sequences, mainly based on myelin basic protein (MBP), by different administration routes (9, 10), as DNA vaccines (11), or in altered forms (12, 13), all showed encouraging results in animal models, but did not perform well in clinical studies in MS patients. More recent approaches aim to harness immune modulatory properties of the innate immune system, particularly through generation of alternatively-activated tolerogenic dendritic cells (DC) and other myeloid antigen-presenting cells (APC) that are essential for peripheral immune tolerance and preventing autoimmunity by limiting effector T cells and inducing regulatory T cells (Treg) (14–17). Encephalitogenic peptides chemically-coupled to syngeneic mouse splenocytes or microparticles inhibit EAE in a peptide-specific manner *via* induction of macrophage-mediated immunomodulatory mechanisms (18, 19), and coupled to autologous human PBMC reduce antigen-specific T cell responses in MS patients (20). Also, mouse and human MHC-peptide constructs treat EAE, and enhance type 2 (“M2”) macrophages and repair in the CNS (21). Direct targeting of T cell antigens to immature DC and macrophages using ligands for C-type lectin receptors such as DEC-205 (16), DCIR2 (22), or mannose receptor (CD206, MR) (23, 24), is another promising approach. Recently, a clinical study in patients with MS and NMOSD showed that intravenous administration of tolerogenic DC loaded with CNS antigens is safe and feasible (25). The therapeutic efficacy of APC targeting approaches in CNS demyelinating diseases remains to be shown.

We previously showed that MOG35-55 conjugated to oxidized mannan polysaccharide (OM-MOG) protects animals against the clinical and pathological features of MOG-EAE in a peptide-specific manner across different MHC class II (MHCII) types in prophylactic and therapeutic applications (24). Protection is associated with the maturation of functionally deficient Th1 and Th17 cells, but the mechanism of tolerance has remained elusive (24). Here we show that OM-MOG both protects against and treats MOG-EAE in humanized HLA-DR2b transgenic mice expressing the human MHCII MS candidate susceptibility genes DRA*0101 and DRB1*1501 (DR2b.Ab° mice) (26, 27). OM-MOG treatment rapidly and almost completely reverses clinical symptoms, reducing inflammatory infiltrates, microglia activation, demyelination, and axon damage in the spinal cord of DR2b.Ab° mice. Supporting studies in B6 mice showed that OM-MOG treatment is associated with a peripheral type 2 myeloid cell response, induction of T cell anergy, preservation of axons within lesions and increased expression of genes associated with recovery of myelin and neurons in the spinal cord. In a Hellenic cohort of MS patients, a high proportion showed peripheral T cell proliferation responses to hMOG35-55, as well as other myelin peptide antigens, across different HLA-DRB1 genotypes. The results suggest that patients with CNS demyelinating diseases in which the autoimmune targets are known might be candidates for peptide-specific therapy with OM-peptides independent of HLA-DRB1 genotype.

## Materials and Methods

### MS Patients, HLA-DRB1 Genotyping, and In Vitro Lymphocyte Proliferation Assay

The protocol for sampling blood from MS patients and healthy individuals for T cell proliferation assays was reviewed and approved by the Ethics committee of the Aeginition Hospital of the National Kapodistrian University of Athens as being consistent with the Declaration of Helsinki (Protocol No: 7BΣH46Ψ8N2-B66, 13/05/2015). The donors signed a written informed consent before donating blood for this study. Considering the core association of the HLA-DRB1*1501 allele with MS risk, clinical course and therapeutic response, including in the Hellenic population (28), we genotyped patients for HLA-DRB1 and included individuals carrying the DRB1*15 allele in our sample (**Table 1**). DNA extraction was performed with the “QIAamp Blood Maxi” commercial kit (QIAGEN, Germany) while DRB1 genotyping was performed using a commercial kit based on the PCR-SSO (Polymerase-Chain-Reaction, Sequence-Specific Oligonucleotide) technique. This method depends on reverse hybridization (Line Probe Assay, INNO-LiPA, Low Resolution, DRB1 Amp Plus, Innogenetics, Fujirebio, Europe) according to the manufacturer’s protocol, for all the specificities included in the HLA Nomenclature of 2012. PBMC were isolated and analyzed for antigen-specific T cell responses by proliferation assay with thymidine incorporation, based on a previously described method (20). Briefly, blood was collected in EDTA-coated tubes, diluted 1:1 with RPMI containing Glutamax and stored at 4°C for 3 days. PBMC were isolated by Biocoll (Biochrom) density gradient centrifugation, seeded in 96-well plates at 1.5 x 10^5^ cells/well in RPMI with 5 μM of each peptide. Forty-eight wells were incubated per peptide, 24 or 48 wells with medium alone as negative control and 6 wells with plate bound anti-CD3 mAb (2 μg/ml) (clone HIT3a; BD Biosciences, San Diego, CA, USA), or phytohaemagglutinin (PHA; 5 μg/ml), as positive control. On day 7 cells were pulsed with 1 μCi/well [^3^H]-thymidine (Amersham Radiochemicals) for 16 h. Thymidine incorporation was measured using a β scintillation counter and cell proliferation was calculated as described above. For each sample, any of the 48 replicas showing CPM higher than the mean + 3 SD of all the unstimulated cells were considered as positive. Peptide-specific responses were considered positive with two or more positive replicas.

**Table 1:** Clinical and demographic data of multiple sclerosis (MS) and healthy control blood donors.

Patient	Age (years)	Sex	MS type/therapy	Disease duration (years)	OCB	Relapse 12/24 mo	MRI Brain/Gd	MRI Spine/Gd	EDSS	HLA-DRB1 low resolution
1	33	F	RRMS Fingolimod	20	+	1/2	+/-	+/-	2.0	03/03
2	48	F	RRMS Interferon-b-1a	13	+	0/2	+/- (↑)	+/- (↑)	3.5	14/15 (DR2)
3	25	M	RRMS Cortisone	New	+	1/1	+/-	+/+	1.5	11/11
4	45	F	RRMS None	New	+	1/2	+/- (↑)	+/+ (↑)	3.0	04/16 (DR2)
7	35	M	RRMS Cortisone	New	–	1/1	+/-	-/-	1.0	04/12
8	57	M	RRMS Interferon-b-1a	25	–	0/0	+/-	+/-	4.0	07/16 (DR2)
9	40	F	RRMS Interferon-b-1a	5	+	3/5	+/-	-/-	4.0	15/16 (DR2)
10	37	F	RRMS Fingolimod	10	+	3/3	+/+(↑)	+/-	3.5	15/16 (DR2)
11	30	F	RRMS Interferon-b-1a	10	+	1/1	+/± (↑)	+/- (↓)	2.5	04/15 (DR2)
17	35	F	RRMS Fingolimod	20	+	1/0	+/- (↑)	+/-	2.0	13/13
18	54	F	OPN	CIS	–	1/0	+/-	+/-	1.0	ND
22	35	M	RRMS Natalizumab	18	+	0/0	+/- (↓)	+/- (↓)	2.5	04/16 (DR2)
N1	23	F	Healthy	NA	NA	NA	NA	NA	NA	04/15 (DR2)
N2	50	F	Healthy	NA	NA	NA	NA	NA	NA	01/04

Key: EDSS, expanded disability status scale; Gd, gadolinium; OCB, oligoclonal bands in cerebrospinal fluid; OPN, optic neuritis; NA, not applicable; ND, not done; RRMS, relapsing-remitting MS; ↑ ↓, increased or decreased lesion load compared to previous MRI, respectively (MRI performed less than 6 months prior to blood sampling); CIS, clinically isolated syndrome.

### Mice

Humanized HLA-DR2b transgenic mice expressing chimeric constructs in which the peptide binding part of mouse I-E are replaced by the corresponding domains of human DR2b molecules (DRA*0101 and DRB*1501), were crossed with mice that lack all mouse MHCII genes, to generate the DR2b.Ab° mice used in this study (26, 27). DR2b.Ab° mice were maintained on a mixed C57BL/6 (B6)/DBA genetic background. MOG-specific T cell receptor (2D2) transgenic mice on a B6 genetic background have been described previously (29) (Tcra2D2, Tcrb2D2)1Kuch/J; The Jackson Laboratory). Inbred B6 mice (Harlan) were used for supporting experiments. Mice were kept under specific pathogen-free conditions in the Department of Animal Models for Biomedical Research of the Hellenic Pasteur Institute. All animal experiments complied with ARRIVE guidelines and were carried out in accordance with the local Ethical Committee guidelines on the use of experimental animals at the Hellenic Pasteur Institute, were approved by the national authorities and conformed to EU Directive 2010/63/EU for animal experiments.

### Synthesis of Peptide Conjugates and Administration in Mice

Peptides MBP13-32, MBP83-99, MOG1-20, mouse and human MOG35-55 (S42 in MOG, P42 in hMOG, respectively), PLP139-154, PLP178-191, and (KG)5-MOG35-55 were synthesized, and (KG)5-MOG35-55 was conjugated to OM as previously described (24, 30, 31). For this study we used OM, in which mannose polysaccharides are converted to polyaldehydes by sodium periodate treatment, and lack complement-activating activity (32). OM-MOG was administered to mice using prophylactic and therapeutic protocols. In the prophylactic protocol, groups of 6- to 8-week-old female DR2b.Ab° or B6 mice were injected intradermally (i.d.) on the flanks with 100 μl of carbonate-bicarbonate buffer pH 9.0 (vehicle) containing OM-MOG (30 μg MOG peptide equivalent/injection and 700 μg OM equivalent/injection) or vehicle control. Three consecutive injections were performed at 15-day intervals, and EAE was induced 15 days after the last injection. In the therapeutic protocol, groups of female DR2b.Ab° or B6 mice were injected as above, starting after disease onset when the clinical score of each individual mouse reached 2 (between days 12-14 post-immunization for EAE), and repeated every 2 days for a total of 5 injections per mouse.

### EAE Induction

MOG-EAE was induced in groups of at least 6- to 8-week-old (for therapeutic administration), or 12- to 14-week-old (after prophylactic administration), female DR2b.Ab° mice, or Ab° control mice, by s.c. tail-base injection of 200 μg MOG in 100 μl saline emulsified in an equal volume of complete Freund's adjuvant (CFA) (Sigma-Aldrich). CFA was supplemented with 400 μg/injection of H37Ra *Mycobacterium tuberculosis* (Difco). In mice injected prophylactically with OM-MOG or vehicle, EAE was induced 15 days after the third vaccine injection. All mice received an identical boost immunization 7 days later (**Table 2**). EAE was also induced in groups of 6- to 8-week-old (for therapeutic administration), or 12- to 14-week-old (after prophylactic OM-MOG administration) female B6 mice by s.c. tail-base injection of 37 μg MOG or 200 μg PLP178-191 (33) in 100 μl saline emulsified in an equal volume of CFA (Sigma-Aldrich). CFA was supplemented with 400 mg/injection of H37Ra *M. tuberculosis* (Difco). All mice, including those boosted by a second immunization, received intraperitoneal (i.p.) injections of 200 ng of *Bordetella pertussis* toxin (PTx) (Sigma-Aldrich) at the time of each immunization and 48 h later. Mice were monitored daily for the clinical symptoms of EAE according to the following scores: 0, normal; 1, limp tail; 2, hind limb weakness; 3, hind limb paralysis; 4, forelimb paralysis; 5, moribund or dead (0.5 gradations represent intermediate scores). Animals from score 4 upwards were euthanized. Notably, therapeutic OM-MOG administration in B6 mice reversed all symptoms of conventional scoring, such that only mild residual neurological defects, such as tail stiffness, were recorded as 0.5. All mice were allowed free access to food and water throughout the experiments.

**Table 2:** Incidence and clinical severity of MOG-EAE in DR2b.Ab mice.

Mouse strain	Peptide	Amount/immunization	Incidence	Mean onset (day p.i.)	Mean peak score
DR2b.Ab°	MBP83-99	250 μg (x2)	0/4	–	–
“	hMOG35-55	150 μg	0/3	–	–
“	“	200 μg	1/8	19	1
“		200 μg (x2)	1/3	20	1
“	MOG35-55	35 μg	0/4	–	–
“	“	100 μg	0/3	–	–
“	“	150 μg	0/3	–	–
“	“	150 μg (x2)	1/3		1
“	“	200 μg	3/9	10.7	2
“	“	200 μg (x2)	10/12	13.1	3
Ab°	“	200 μg (x2)	0/3	–	–

### Mouse T Cell Proliferation Assays

For [^3^H]-thymidine incorporation, splenocytes and draining lymph node (DLN) cells were recovered and cultured for 120 h in RPMI 1640 (Sigma-Aldrich) supplemented with 10% heat-inactivated FBS, 50 μM 2-mercaptoethanol (Sigma-Aldrich), and increasing concentrations of MOG. Cells were stimulated with MOG in triplicate or quadruplicate at 1 x10^6^ cells/ml in round-bottom 96-well plates (Costar), pulsed with 1 μCi/5x10^5^ cells [^3^H]-thymidine (Amersham Radiochemicals) for the last 16 h of culture. Control cells were stimulated in duplicates with medium alone as negative control or with plate bound anti-CD3 (0,5 μg/ml) (clone 145- 2C11, BD Biosciences, San Diego, CA, USA) as positive control and pulsed with [^3^H]-thymidine as above. [^3^H]-thymidine incorporation was measured using a β scintillation counter. Results are expressed as radioactivity cpm, or the stimulation index (SI) calculated from the cpm of cells cultured in the presence of peptide divided by cpm of cells cultured in medium alone. For CFSE-labeling, splenocytes were labeled with carboxyfluorescein succinimidyl ester (CFSE) (CFDA-SE; Thermofisher Scientific). Cells were washed, resuspended at 10^7^/ml in PBS in 24-well plates, and CFSE was added at a final concentration of 5 μM for 9 min at room temperature (RT). Cells were washed with PBS containing 2% FBS to stop the reaction, resuspended in RPMI 1640 (Invitrogen Life Technologies) supplemented with 10% heat-inactivated FBS, 50 μM 2-mercaptoethanol (Sigma-Aldrich), at 10^6^/ml, and stimulated with 15 μg/ml MOG or PLP178-191 peptide for 72 h. Undivided cells showed strong CFSE fluorescence and were designated “CFSE high”, while divided cells progressively lose CFSE fluorescence and were collectively designated “CFSE low”.

### FACS

For cell markers and cytokine production, splenocytes and DLN cells were recovered and stimulated *in vitro* with MOG, PLP178-191 or plate-bound anti-CD3 antibody (0.5 ug/ml; clone 145-2C11, BD Biosciences) as above. For cell surface markers, Fc receptors were blocked using antibody to CD16/CD32 (mouse Fc Block^™^; clone 2.4G2; BD Biosciences), cells were fixed in 2% paraformaldehyde solution in PBS (PFA) for 20 min at 4°C and stained with antibodies to surface markers, CD4 (clone L3T4; BD Biosciences), CD44 (clone IM7; BD Biosciences), CD11b (clone M1/70; BD Biosciences), CD11c (clone HL3; BD Biosciences), PD-L1 (clone MIH5; BD Biosciences), and CD25 (clone PC61.5; BD Biosciences). For intracellular staining, cells were stimulated for 3 h with PMA (10 ng/ml) and ionomycin (1 μg/ml) in the presence of brefeldin A (5 μg/ml), fixed in 2% PFA as above, permeabilized with 0.5% wt/vol saponin and stained with antibodies to IL-17 (clone TC11-18H10; BD Biosciences), IFN-γ (clone XMG1.2; BD Biosciences), IL-5 (clone TRFK5; Affymetrix eBioscience), IL-2 (clone JES6-5H4; BioLegend), IL-10 (JES5-16E3; BD Biosciences), FoxP3 (FJK-16s; Thermofisher Scientific), and FR4 (12A5; Biolegend). Peptide-loaded bone marrow-derived DC were fixed in 2% PFA as above and stained with antibodies to CD11c (clone HL3), CD40 (clone 3/23), and CD80 (clone 16-10A1) (all BD Biosciences). Data was acquired with a FACS Calibur cytometer and analyzed with CellQuest (BD) and FlowJo (Tree Star, Inc) software. To measure IL-2 and CD25 production, the specific signal was determined as the increase in geometric mean of staining and expressed as mean fluorescence intensity (MFI).

### Dendritic Cell Isolation and Antigen Presentation Assays

Bone marrow-derived DC were generated from DR2b.Ab° or B6 mice based on a previously described method (24). Briefly, bone marrow cells were cultured at 5 x 10^6^ cells per plate in 10 ml complete RPMI supplemented with recombinant granulocyte-macrophage colony-stimulating factor (GM-CSF) (20 ng/ml) (Peprotech) for 9 days. Cells were loaded with OM-MOG, MOG, OM or PBS (10 or 20 μg/ml peptide equivalent and 233 or 466 μg/ml mannan equivalent for B6 or DR2b.Ab° DC, respectively), or stimulated with lipopolysaccharide (LPS) (1 μg/ml) derived from *Escherichia coli* (Sigma) as positive control for DC maturation. After 24 h the cells were used for DC-T cell antigen presentation assays or phenotyped for surface markers and analyzed by FACS as described above. For DC-T cell co-culture assays, splenocytes and DLN cells isolated from non-immunized 2D2 MOG-specific TCR transgenic mice (29) were co-cultured at 2 x 10^5^ cells/well in 96 well plates with peptide-loaded DC at a 5:1 ratio in triplicate for 120 h (5 days) in the absence or presence of IL-2 (10 ng/ml, Peprotech).

### Histopathological Analysis

Mice were transcardially perfused with ice-cold 4% PFA at sacrifice by carbon dioxide inhalation. The vertebral column was dissected and post-fixed in the same fixative for 16 h at 4°C, spinal cord was removed, embedded in paraffin and processed for standard histopathological analysis. Inflammation was visualized by haematoxylin and eosin (H&E), demyelination by Luxol fast blue (LFB)/periodic acid-Schiff staining, axon damage by Bielschowsky’s silver stain. Immunohistochemistry was performed using anti-amyloid precursor protein (APP) (1/300, Merck Millipore: MAB348), and anti-Iba1 (1/500, Wako: 019-19741) antibodies to measure axon damage and microgliosis, respectively, as previously described (34). Inflammation was determined by semi-quantitative scoring as follows: 1: foci of subarachnoid cell infiltration or meningeal inflammation, 2: diffuse subarachnoid infiltration or perivascular infiltration, 3: foci of parenchymal infiltration, 4: diffuse widespread parenchymal infiltration. Demyelination was evaluated by semi-quantitative scoring as follows; 0.5: single perivascular sleeves of demyelination, 1: ubiquitous perivascular or sub-pial demyelination, 2: confluent demyelinated plaques, 3: profound focal demyelination, involving about 1/2 of the spinal cord white matter at least in one spinal cord segment, 4: extensive demyelination, for instance complete demyelination of spinal cord white matter in one or more segment of cord. Images were scored blindly by two independent observers. Microglia activation was measured as the % area covered by Iba1-immunoreactivity. APP spheroids were counted in 3–4 fields/image at 20x magnification and expressed as APP spheroids/mm^2^.

### Electron Microscopy

Ultrastructural analysis of spinal cord lesions was performed according to previously described methods (35). B6 mice were perfused with 4% paraformaldehyde/2.5% glutaraldehyde in phosphate buffer pH7.4, and 300 μM transverse slices of spinal cord were post-fixed in the same fixative. The region of interest was punched out, postfixed in 2% osmium tetroxide (Science Services) and embedded in Epon (Serva). Ultrathin sections (50 nm) were collected onto copper grids (Gilder Grids Ltd.), coated with formvar (Plano), and stained with UranylLess^®^ (Science Services). Electron micrographs of cross-sectioned axons in the anterior funiculus of lumbar spinal cord, a primary site of inflammatory lesions, were taken with a LEO EM912 omega electron microscope (Zeiss) using an on-axis 2k CCD camera (TRS). Twelve random pictures (at 3150x) were taken as multiple image assemblies from each mouse (5x5 tiles per image covering an area of 33µm x 33µm each). For the analysis of axon morphology, all fibers per picture, using all 12 pictures, were manually counted and evaluated for maintenance or loss of normal axon morphology using Image J (NIH) software. For the analysis of myelin, g-ratios were calculated from 80 fibers per picture, using 5 pictures per mouse. On each micrograph, fiber diameter and axon diameter of each fiber were manually measured, taking an average from 3 diameters per element. Three mice per group per time point were analyzed. Data are expresses as percentages and numbers of myelinated and collapsed axons, and g-ratios (axon diameter/fiber diameter) for all myelinated axons measured in the spinal cord of each mouse in each group.

### Quantitative RT-PCR Analysis

Spinal cords were flushed from the vertebral column after transcardial perfusion with ice-cold PBS at sacrifice by carbon dioxide inhalation. Total RNA was extracted from spinal cords and spleens using TRIzol reagent (Invitrogen, Paisley, U.K.), according to the manufacturer’s instructions. RNA purity and quantification were assessed using NanoDrop 2000 spectrophotometer data. For cDNA synthesis, 500ng of total DNase-treated RNA was reverse transcribed using the SuperScript™ First-Strand Synthesis System (Invitrogen) according to the manufacturer’s instructions. The cDNA was used as template for quantitative PCR using a SYBR™ Select Master Mix (Applied Biosystems), according to the manufacturer’s instructions. All reactions were performed in duplicate using the LightCycler system (Roche, Mannheim, Germany). At the end of each PCR run, melting curve analysis was performed to verify the integrity and homogeneity of PCR products. All results were analyzed using LightCycler software version 3.5 (Roche). QuantiTect Primer Assays (QIAGEN) were used for *H2Ab1* (Mm_H2-Ab1_1_SG), *Mbp* (Mm_Mbp_1_SG), *Snap25* (Mm_Snap25_2_SG), *Mrc1* (Mm_Mrc1_1_SG), *Mmp9* (Mm_Mmp9_1_SG), *IL1β* (Mm_Il1b_2_SG), *Chi3I3* (Mm_Chi3l3_1_SG), and *Gusb* (Mm_Gusb_1_SG). Gene-expression analyses were performed using *Gusb* as a reference gene. The difference between threshold cycle values of the target and reference gene (ΔCT = Ct target gene - Ct reference gene) in each sample was calculated and the relative gene expression was estimated according to the ΔΔ threshold cycle method in which ΔΔCT = (ΔCT of a target sample) - (ΔCT of the average of reference/naïve samples). The final relative gene expression was expressed as 2^–ΔΔCT^ value.

### Statistical Analysis

All statistical analyses were performed with Sigma Stat 3.5, Sigma Plot 11, GraphPad Prism 5 and Microsoft Excel. At experimental time points where low numbers of mice were used (**Figure 7**), data were first checked for normal distribution using the Shapiro-Wilk normality test prior to statistical analysis. All data are given as mean ± SEM. Student’s t test, one-way (DC-T cell assays), and two-way (EAE scoring) ANOVA tests were used, followed by Bonferroni post-hoc tests, or Kruskall-Wallis test (electron microscopy analyses). Results were considered statistically significant when *p ≤* 0.05.

## Results

### OM-MOG Both Prevents and Treats MOG-EAE in DR2b.Ab° Mice

**Figure 1:**
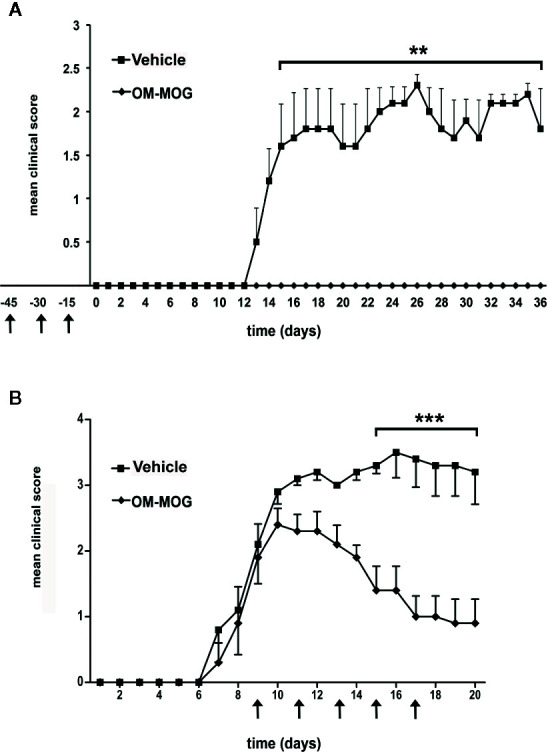
Both prophylactic and therapeutic administration of OM-MOG protect DR2b.Ab° mice against the clinical symptoms of MOG-EAE. Mean clinical scores of MOG-EAE in DR2b.Ab° mice injected i.d. with OM-MOG or vehicle control at indicated time points (arrows) using a prophylactic **(A)** or therapeutic protocol **(B)**. EAE was induced by two immunizations (days 0 and 7) with MOG/CFA/PTx (prophylactic administration: OM-MOG mice n=4, vehicle n=5; therapeutic administration: OM-MOG mice n=5, vehicle n=5). One moribund animal in the therapeutic vehicle group **(B)** was euthanized on day 16 and was given a clinical score of 5 for the remaining days of the experiment. Data from one **(B)** or one representative of two **(A)** independent experiments are shown. Statistical significance after comparisons between groups of mice using Student’s *t* test is shown (**p ≤ 0.01, ***p ≤ 0.001).

Humanized HLA-DR2b transgenic mice lacking all mouse MHCII genes (DR2b.Ab° mice) were used for investigating the tolerogenic effects of OM-MOG in MOG-EAE. MOG, hMOG, and MBP87-99 are human DR2-restricted myelin determinants, with MOG being strongly immunogenic and encephalitogenic in DR2b.Ab° mice (27). First, we established a reproducible EAE protocol in DR2b.Ab° mice by immunizing them with different amounts of MBP83-99, MOG, or hMOG, which differs from MOG by a single residue (S42 in MOG, P42 in hMOG), with or without an identical boost immunization 7 days later, each accompanied by PTx injections. Both MOG peptides induced EAE, but only mice receiving two immunizations of 200μg MOG consistently developed EAE with high incidence and reproducible clinical characteristics, and this protocol was chosen for experiments (**Table 2**) (**Figure 1**). Consistent with a previous report for MBP87-99 (27), MBP83-99 failed to induce EAE in DR2b.Ab° mice lacking a human MBP-specific TCR. As expected, control Ab° littermates lacking the HLA-DR2b transgene did not develop MOG-EAE, confirming that MOG presented exclusively in the context of human HLA-DR2b is sufficient to activate mouse MOG-specific T cells and to induce EAE (**Table 2**). DR2b.Ab° mice showed less susceptibility to MOG-EAE than B6 mice (24), probably due to the mixed genetic background of DR2b.Ab° transgenic mice (B6/DBA), and because MOG is presented to MOG-specific T cells exclusively in the context of human HLA-DR2b, not mouse MHCII molecules.

To examine the effect of OM-MOG upon the development of EAE, we injected groups of DR2b.Ab° mice using prophylactic and therapeutic administration protocols as previously described (24). In the prophylactic protocol, mice received three i.d. injections of OM-MOG, in dilute soluble form and spaced at 15-day intervals prior to immunization with MOG/CFA/PTx. Mice were completely protected against the development of EAE, compared to full-blown EAE that developed in vehicle controls (**Figure 1A**). In the therapeutic protocol, mice received i.d. injections after the onset of MOG-EAE, starting when they reached score 2, and repeated every 2 days for a total of 5 injections per mouse. The clinical score rapidly reduced, and overall well-being in OM-MOG-treated mice was obvious from one day after the first injection of OM-MOG, compared to full-blown EAE that developed in vehicle controls (**Figure 1B**). Long term effects of OM-MOG therapy were not studied in DR2b.Ab° mice here, but in a previous study with B6 mice therapeutic OM-MOG showed continuous beneficial effects up to the last time point studied (37 days post-immunization for EAE, 25 days after starting treatment with 4 weekly injections) (24).

### OM-MOG Prevents and Reverses Neuropathological Lesions of MOG-EAE, Preserves Axon Integrity in Spinal Cord Lesions and Increases Gene Expression Associated With Myelin and Neuron Recovery

**Figure 2:**
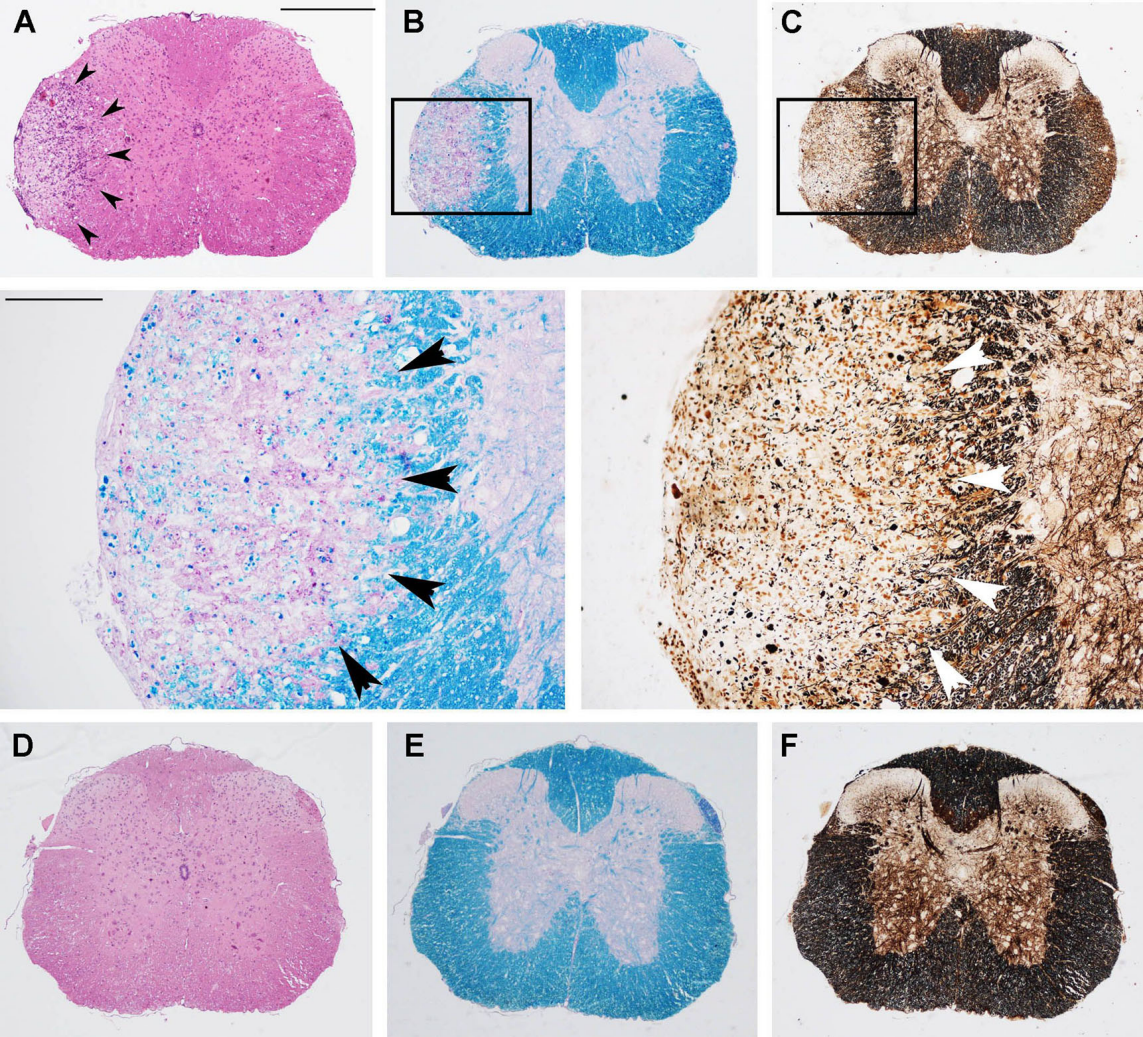
Prophylactic OM-MOG prevents the development of spinal cord neuropathology during MOG-EAE in DR2b.Ab° mice. Neuropathological analysis of spinal cord sections from prophylactic vehicle- (upper and middle panels) and OM-MOG-injected (lower panels) DR2b.Ab° mice on day 36 post-immunization for EAE. Inflammatory cell infiltration was visualized by H&E **(A, D)**; demyelination by Luxol fast blue [**(B)** and enlarged inset, **(E)**]; axon damage by Bielschowsky’s silver staining [**(C)** and enlarged inset, **(F)**]. Vehicle-treated mice show large confluent inflammatory, demyelinating lesions with axon damage typical of MOG-EAE (arrowheads). OM-MOG-vaccinated mice showed no spinal cord pathology. Scale bars 500 μM **(A–F)**, 100 μM enlarged inserts in middle panels. Representative sections from 1 of 4 (OM-MOG) or 5 (vehicle) mice per group are shown.

**Figure 3:**
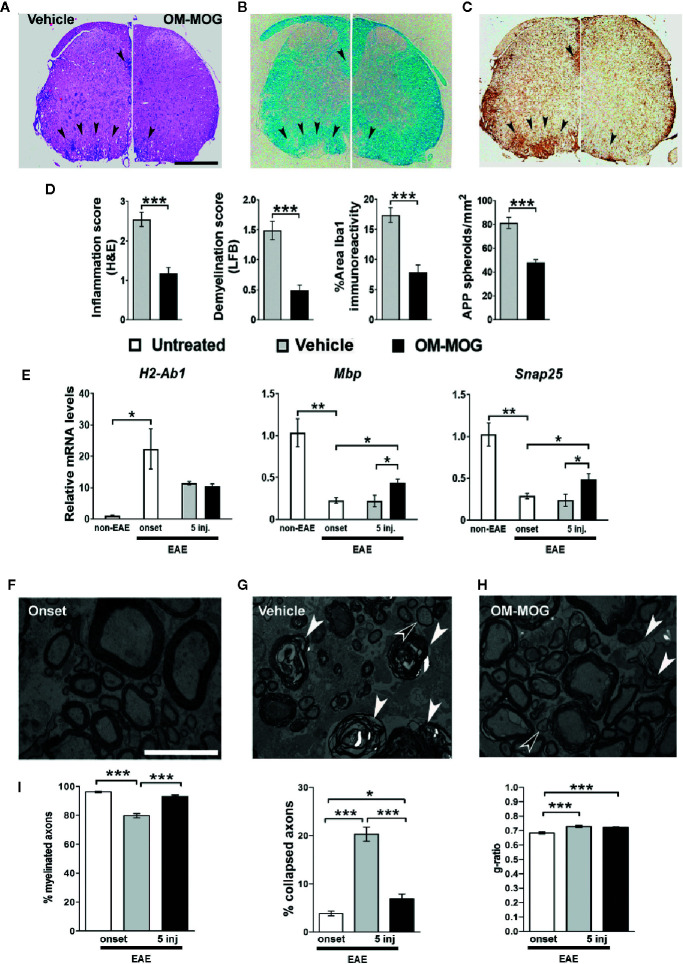
Therapeutic OM-MOG reverses spinal cord neuropathology in DR2b.Ab° mice, and increases the expression of myelin and neuronal genes, and preserves axons in B6 mice during MOG-EAE. **(A–D)** Neuropathological analysis of spinal cord sections from therapeutic vehicle- (left panels of each photo) and OM-MOG-injected (right panels of each photo) DR2b.Ab° mice on dpi 20 for EAE. **(A, D)** Inflammatory cell infiltration was measured by H&E; **(B, D)** demyelination by LFB; **(C, D)** microgliosis by Iba1 immunostaining; **(D)** axon damage by amyloid precursor protein (APP) immunostaining. Vehicle-treated control mice show large confluent inflammatory, demyelinating lesions with neuroinflammation typical of MOG-EAE (arrowheads). OM-MOG-treated mice showed markedly reduced spinal cord pathology. Representative sections from 1 of 5 (OM-MOG) or 4 (vehicle) mice per group are shown. Scale bar 500 μM. **(E)** Quantitative RT-PCR analysis of mRNA levels for selected markers, *H2-Ab1* (inflammation), *Mbp* (myelin) and *Snap25* (neurons), relative to *Gusb*, in whole spinal cord recovered from B6 mice before immunization for MOG-EAE (non-EAE, n=4) or during MOG-EAE on the day of the first clinical symptom (onset, n=4) and after 5 injections of vehicle (n=5; for *H2-Ab1*, n=4) or OM-MOG (n=5; for *H2-Ab1* n=4). **(F–H)** Representative electron micrographs of anterior funiculus of lumbar spinal cord, a major site of inflammatory lesions, from B6 EAE mice before treatment (clinical score 2, “onset”) **(F)**, or after 5 i.d. injections of vehicle **(G)** or OM-MOG **(H)**. White arrowheads show collapsed axons, black arrowheads show remyelinated axons, scale bar 5 μM. **(I)** Percentages of non-collapsed myelinated axons (left graph) and collapsed axons (middle graph), and quantification of the g-ratios (axon diameter/fiber diameter) of myelinated axons (right graph) in the same groups of mice shown in **(F–H)** (n=3 per group). Therapeutic effects of OM-MOG at the clinical level were seen in one (DR2b.Ab° mice) or more (B6) independent ËAE experiments. Data and statistical significance for each analysis are derived from one experiment each, after pairwise comparisons between samples from different mouse groups using Student’s t test **(D, E)**, Kruskal-Wallis test (**I**, axon measurements) or one-way ANOVA (**I**, g-ratios) (*p ≤ 0.05, **p ≤ 0.01 ***p ≤ 0.001).

To determine whether amelioration of the clinical signs of MOG-EAE by OM-MOG in DR2b.Ab° mice was associated with reduced neuropathology, prophylactic OM-MOG or vehicle mice were sacrificed 36 days after EAE induction for neuropathological analysis. As in B6 mice (24), vehicle DR2b.Ab° mice showed large confluent lesions with mononuclear cell infiltration, demyelination and axonal damage in the spinal cord white matter (**Figure 2**, upper and middle panels), while OM-MOG mice showed very low levels of inflammatory cell infiltration, mainly meningeal, and no demyelination or axonal damage (**Figure 2**, lower panels). Therapeutic OM-MOG and vehicle DR2b.Ab°mice were sacrificed 20 days after EAE induction for neuropathological analysis. Therapeutic OM-MOG mice showed reduced CNS inflammation, as shown by mononuclear cell infiltration (**Figures 3A, D**) and Iba1-immunostaining of macrophages/microglia (**Figures 3C, D**), demyelination (**Figures 3B, D**), and axonal damage (**Figure 3D**), compared to vehicle mice.

To assess whether therapeutic OM-MOG promotes CNS recovery, to overcome difficulties in obtaining sufficient numbers of DR2b.Ab° mice we treated MOG-EAE in B6 mice with OM-MOG. Spinal cord recovered from therapeutic OM-MOG mice after 5 injections of OM-MOG showed increased expression of myelin (*Mbp*) and neuronal (*Snap25*) marker genes compared to vehicle controls and importantly, significantly increased expression compared to untreated mice at onset, suggesting that OM-MOG actively promotes both myelin and neuron recovery (**Figure 3E**).

To investigate possible neuroprotective effects of OM-MOG treatment, we performed electron microscopy analysis of lesions in the white matter of the anterior funiculus of lumbar spinal cord taken from groups of EAE mice before treatment (mice reaching clinical score 2) (**Figure 3F**), and after 5 injections of vehicle (**Figure 3G**) or OM-MOG (**Figure 3H**). The EAE pretreatment group showed loosened myelin sheaths while axons remained well preserved with homogenous low electron dense axioplasm (**Figure 3F**). Significant damage was evident after vehicle treatment, where vacuolation, blebbing and unraveling of the myelin occurred, and many of the enveloped axons were either undistinguishable or electron dense, both signs of axon collapse (**Figure 3G**, white arrowheads show collapsed nerve fibers). In contrast, after OM-MOG treatment, tissue ultrastructure was similar to that in the pretreated mice, with loosened myelin sheaths and preservation of most axons, although some collapsed axons (white arrowheads) and vacuoles separating myelin/axon contact could be seen (**Figure 3H**). Quantitative analysis of axon profiles showed that the percentages (**Figure 3I**) and numbers (**Supplementary Figure 1A**) of non-collapsed myelinated axons were higher in OM-MOG compared to vehicle mice, and equal to those in the pre-treatment EAE onset group. Conversely, the percentage (**Figure 3I**) and numbers (**Supplementary Figure 1B**) of collapsed axons was significantly lower in OM-MOG compared to vehicle mice, closer to those in the pre-treatment EAE onset group.

We next investigated whether OM-MOG treatment has an effect on remyelination, which indirectly protects axons. Nerve fibers in vehicle and OM-MOG mice showed equal increases in g-ratios compared to pretreatment EAE onset mice (**Figure 3I**), suggesting that remyelination occurs equally in the lesions of both groups of mice. This observation indicates that remyelination might proceed independently of ongoing inflammation in the vehicle control mice. Together, these results show that OM-MOG prevents and treats MOG-EAE in DR2b.Ab° and B6 mice, reversing clinical symptoms and inhibiting inflammatory demyelination, preserving the integrity of axons within the lesions and increasing the expression of genes associated with myelin and neuron recovery in the spinal cord.

### OM-MOG Tolerized T Cells in DR2b.Ab° Mice Show Impaired MOG-Specific Proliferation Responses and IL-2 Production

**Figure 4:**
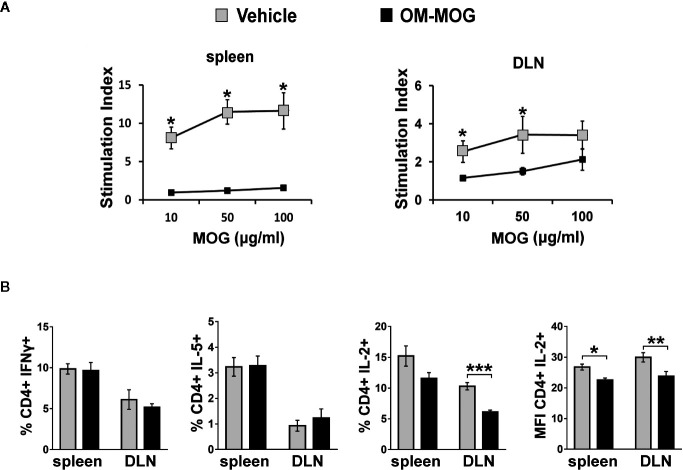
OM-MOG tolerized T cells in DR2b.Ab° mice show impaired myelin oligodendrocyte glycoprotein (MOG)-specific proliferation responses and IL-2 production. **(A)** Proliferation of splenocytes and draining lymph node (DLN) cells isolated from prophylactic DR2b.Ab° mice dpi 13 with MOG/CFA (n=5 per group). Cells were stimulated *in vitro* with MOG and thymidine incorporation was measured. **(B)** FACS of CD4^+^ T cells among splenocytes and DLN cells isolated from prophylactic DR2b.Ab° mice dpi 13 with MOG/CFA, for production of IFN-γ, IL-5 and IL-2, measured by intracellular cytokine staining (n=5 per group). Data and statistical significance are derived from one (IL-5), or one representative of three independent experiments. Statistical significance after pair-wise comparisons between cells from different mouse groups using Student’s *t* test is shown (*p ≤ 0.05, **p ≤ 0.01, ***p ≤ 0.001).

To determine whether the beneficial effects of OM-MOG in DR2b.Ab° mice were associated with altered antigen-specific immune responses, we compared T cell responses in mice receiving prophylactic OM-MOG or vehicle and then immunized with MOG. DLN cells and splenocytes were recovered at different time-points after the first immunization, re-stimulated with MOG peptide *in vitro* and analyzed for proliferation, expression of activation markers and effector cytokine production. As in prophylactic OM-MOG B6 mice (24), antigen-specific T cell proliferation responses in prophylactic OM-MOG DR2b.Ab° mice, dpi 13 with MOG/CFA (for T cell priming) (**Figure 4A**), or 17 and 36 dpi with MOG/CFA/PTx (for MOG-EAE), were consistently reduced compared to cells from vehicle controls. Also, as in B6 mice, MOG-specific Th1 (IFN-γ-producing) and Th2 (IL-5-producing) cells expanded equally in prophylactic OM-MOG and vehicle DR2b.Ab° mice showing no alteration of effector T cell maturation or overt Th1/Th2 immune deviation by OM-MOG (**Figure 4B**). However, MOG-specific IL-2-producing CD4^+^ T cells were significantly reduced in the spleen and DLN of prophylactic OM-MOG DR2b.Ab° mice (**Figure 4B**). MOG-specific IL-17-producing T helper cell responses were not detectable in prophylactic OM-MOG or vehicle DR2b.Ab° mice (data not shown).

### OM-MOG DC Stimulate Reduced Proliferation Responses and Production of IL-2 and CD25 by MOG-Specific T Cells

**Figure 5:**
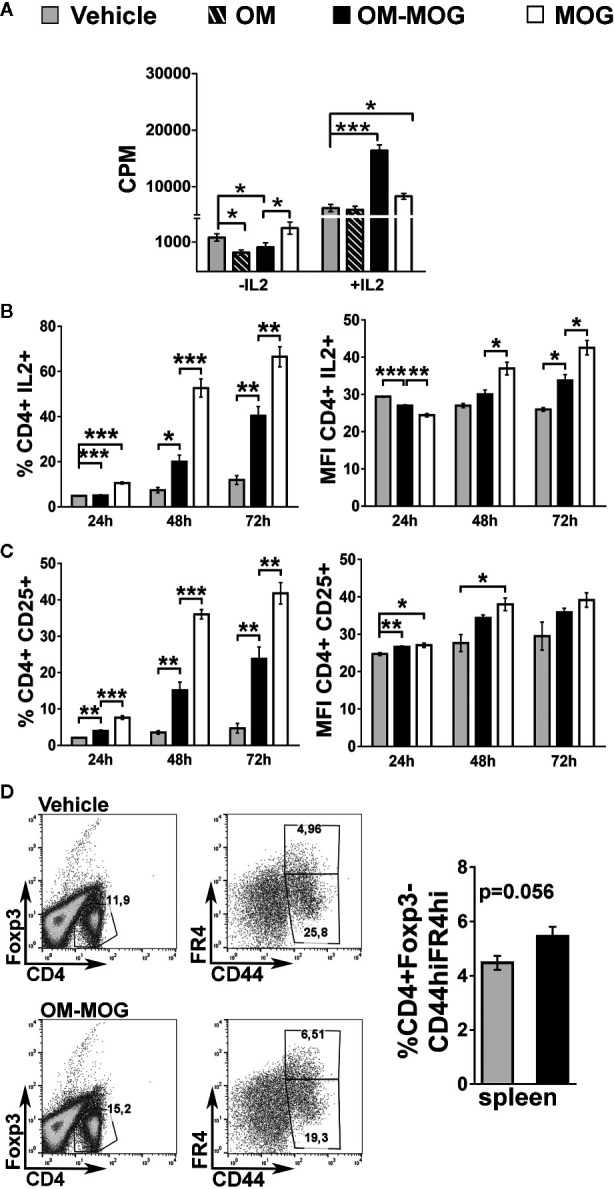
OM-MOG dendritic cells (DC) stimulate reduced proliferation responses and production of IL-2 and CD25 by MOG-specific CD4+ T cells. **(A–C)** *In vitro* antigen presentation assays between peptide loaded DC and 2D2 MOG-specific T lymphocytes. **(A)** Proliferation responses of 2D2 lymphocytes to peptide-loaded DR2b.Ab° DC, in the absence or presence of IL-2, measured by thymidine incorporation. **(B, C)** Flow cytometry of CD4^+^ 2D2 T cell responses to peptide-loaded B6 DC at different culture time points (T cells from n=3 2D2 mice/time point), for production of IL-2 **(B)** and CD25 **(C)**, and represented as proportions (left graphs) and mean fluorescence intensity (MFI) (right graphs) of cells. **(D)** FACS of CD4^+^Foxp3^-^ T cells among splenocytes isolated from therapeutic B6 mice (OM-MOG n=6; vehicle n=5), dpi 10 with MOG/CFA, for surface levels of CD44 and folate receptor 4 (FR4). Data and statistical significance are from one representative of two **(D)**, or three to four independent experiments. Statistical significance after multiple comparisons between groups using Student’s *t* test **(D)** or one-way ANOVA **(A–C)** is shown (*p ≤ 0.05, **p ≤ 0.01, ***p ≤ 0.001).

To test whether reduction of IL-2 signaling and proliferation responses in MOG-specific T cells might underlie the beneficial effects of OM-MOG in EAE, we modeled the situation in DR2b.Ab° mice *in vitro* using antigen cross-presentation assays between bone marrow-derived DC from DR2b.Ab° mice, and I-Ab-restricted MOG-specific splenocytes from 2D2 transgenic mice (29), and measured T cell proliferation responses as read-out. The analysis of DC maturation markers by peptide-loaded, CD11c^+^-gated DC generated from DR2b.Ab° mice showed that OM-MOG DC have an intermediate state of maturation between those of immature (PBS or MOG) and fully matured (LPS or OM) DC (**Supplementary Figure 2**). We compared the effects of DR2b.Ab° DC loaded with MOG, OM, OM-MOG, or PBS in cross-stimulating B6 2D2 MOG-specific T cells, by measuring antigen-specific proliferation responses. Under these conditions, MOG induced a small non-significant increase in T cell proliferation, while both OM and OM-MOG DC reduced T cell proliferation (**Figure 5A**). However, addition of exogenous IL-2 to the cultures induced MOG-specific T cell proliferation responses to both MOG and OM-MOG DC, not to OM DC, compared to PBS DC (**Figure 5A**). These results suggest that OM-MOG presentation by DR2b.Ab° DC stimulates reduced MOG-specific T cell proliferation due to reduced production and/or responsiveness of T cells to IL-2.

To further investigate the effects of OM-MOG on IL-2 signaling in MOG-specific effector T cells we performed antigen presentation assays under matched conditions using B6 DC and B6 2D2 T cells. Both MOG and OM-MOG B6 DC induced increasing production of IL-2 and surface levels of IL-2 receptor (CD25), by CD4^+^ T cells over time in culture, but responses to OM-MOG DC were significantly reduced compared those to MOG DC (**Figures 5B, C**). These results show that presentation of OM-MOG to MOG-specific TCR on CD4^+^ T cells stimulates reduced proliferation, IL-2 and IL-2R production compared to MOG, features that are consistent with the induction of antigen-specific T cell anergy. Further support for OM-MOG-induced T cell anergy is given by the finding of increased proportions of antigen-specific CD4^+^ FoxP3^-^ splenocytes producing high levels of folate receptor 4 (FR4) (CD4^+^FoxP3^-^CD44^hi^FR4^hi^) in prophylactic OM-MOG B6 mice (**Figure 5D**). High expression of FR4 and CD73 are recently-described features shared by anergic CD4^+^ T cells and Treg where both molecules are thought play functional roles in the maintenance of immune tolerance during pregnancy and in individuals susceptible to autoimmune diseases (36).

### Protection From EAE by OM-MOG Is Peptide-Specific

**Figure 6:**
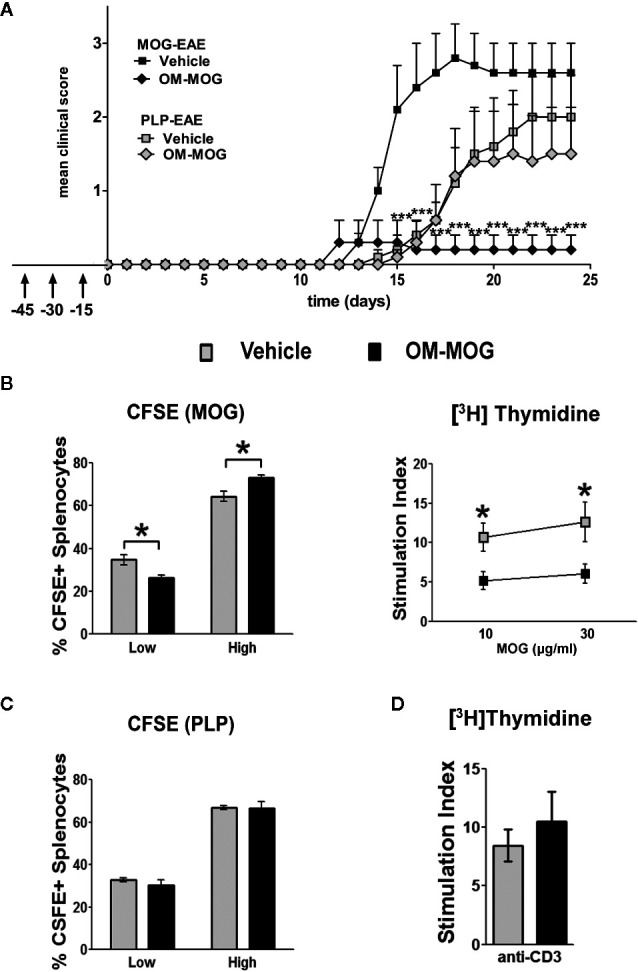
OM-MOG protection against experimental autoimmune encephalomyelitis (EAE) is peptide-specific. **(A)** Mean clinical scores of MOG-EAE and PLP-EAE in B6 mice injected i.d. with OM-MOG or vehicle at indicated time points (arrows) using a prophylactic protocol. EAE was induced by immunization with MOG/CFA/PTx (black symbols) or PLP/CFA/PTx (gray symbols) (MOG-EAE: OM-MOG n=5, vehicle n=5; PLP-EAE: OM-MOG n=5, vehicle n=5). **(B)** Proliferation of splenocytes isolated from MOG-EAE mice shown in **(A)**, dpi 24 (n=5 per group), after stimulation *in vitro* with MOG and flow cytometry for CFSE staining (left) or measurement of thymidine incorporation (right). **(C)** Proliferation of splenocytes isolated from PLP-EAE mice shown in **(A)**, dpi 24 (n=5 per group), after *in vitro* stimulation with PLP and flow cytometry for CFSE staining (left). Undivided cells showed strong CFSE fluorescence and were designated “high”, while divided cells progressively lose CFSE fluorescence and were collectively designated “low”. **(D)** Proliferation of splenocytes isolated from MOG-EAE mice shown in **(A)**, dpi 24 (n=5 per group), after *in vitro* stimulation with plate bound anti-CD3 antibody and measurement of thymidine incorporation. Statistical significance after comparisons between groups of mice using Student’s *t* test **(B–D)** or two-way ANOVA **(A)** is shown (*p ≤ 0.05, ***p ≤ 0.001).

We previously showed that PLP139-151-induced EAE in SJL/J mice is prevented by prophylactic OM-PLP139-151 but not OM-MBP83-99, and that MOG-EAE in B6 mice is prevented by OM-MOG but not OM-PLP178-191, demonstrating peptide specificity of OM-peptide tolerance (24). Here we further show the peptide specificity of OM-peptide tolerance, by investigating whether prophylactic OM-MOG inhibits the ability of another autoantigen to induce autoimmune disease in the same animal, or the induction of polyclonal T cell responses *in vitro*. For this we used two different EAE models in B6 mice, induced by immunization with MOG and PLP178-191 (33). PLP178-191 induced EAE with onset of clinical symptoms a few days later (d14) than those with MOG (d11-12) (**Figure 6A**). Groups of mice were injected with OM-MOG or vehicle, using the prophylactic protocol, and then immunized with MOG or PLP178-191 for the induction of EAE. As predicted, prophylactic OM-MOG inhibited MOG-EAE but not PLP178-191-EAE, which developed into full-blown disease equal to that in the PLP178-191-EAE disease control group (**Figure 6A**).

Clinical results were confirmed in T cell proliferation assays using splenocytes in CFSE labeling and thymidine incorporation assays. Splenocytes from OM-MOG-treated MOG-EAE mice showed reduced proliferation compared to cells from vehicle-treated MOG-EAE mice, in response to MOG stimulation, shown by dilution of CFSE staining (**Figure 6B**, left) and reduced thymidine incorporation (**Figure 6B**, right). In contrast, splenocytes from OM-MOG-treated PLP-EAE mice showed equal proliferation compared to cells from vehicle-treated PLP-EAE mice, in response to PLP stimulation, shown by CFSE staining (**Figure 6C**). To further test whether OM-peptides alter T cell responses to polyclonal stimuli, splenocytes from OM-MOG-treated MOG-EAE mice were stimulated *in vitro* with plate-bound anti-CD3 antibody. Cells from OM-MOG- and vehicle-treated mice proliferated equally in response to anti-CD3 antibody stimulation (**Figure 6D**). These results confirm that OM-peptides induce peptide specific tolerance in mice without affecting T cell responses to other antigens or polyclonal T cell stimuli.

### OM-MOG Induces a Type 2 Myeloid Cell Response in the Periphery

**Figure 7:**
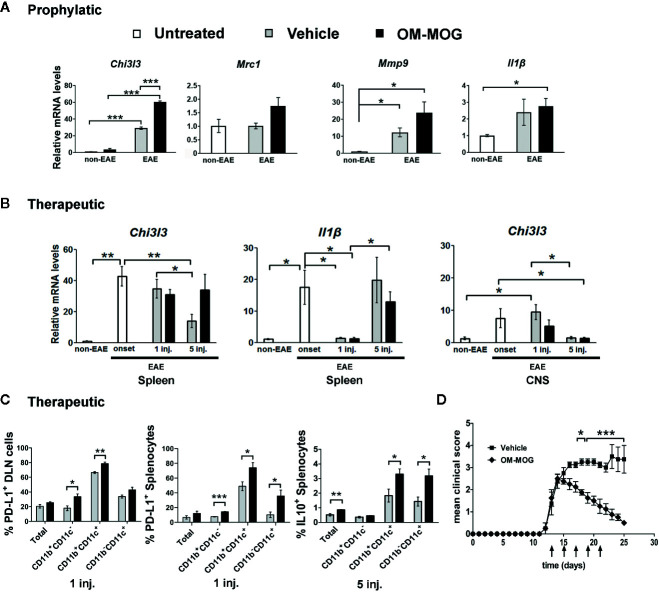
OM-MOG induces a peripheral type 2 myeloid cell response during experimental autoimmune encephalomyelitis (EAE). **(A)** Quantitative RT-PCR analysis of mRNA levels for *Chi3l3*, *Mrc1*, *Mmp9*, and *Il1β* in spleen recovered from B6 mice after prophylactic injections of OM-MOG or vehicle (*Chi3l3*) or from naïve B6 mice (*Mrc1*, *Mmp9*, and *Il1β)* before EAE (non-EAE) and from B6 mice after prophylactic injections of OM-MOG or vehicle dpi 18 after immunization with MOG/CFA/PTx for induction of MOG-EAE (n=3 per group). **(B)** Quantitative RT-PCR analysis of mRNA levels for *Chi3l3* and *Il1β* in spleen and spinal cord recovered from naïve (non-EAE) B6 mice or MOG-EAE B6 mice at different time points before (at clinical score 2, “onset”) or after 1 and 5 therapeutic injections of OM-MOG or vehicle (n=4 per group). **(C)** Flow cytometry of CD11b^+^ and CD11c^+^ myeloid cells among splenocytes and draining lymph node (DLN) recovered from MOG-EAE B6 mice after one and five therapeutic injections of OM-MOG or vehicle, dpi 14 and 25 respectively, for cell surface PD-L1 (left and middle graphs) or intracellular IL-10 production (right graph) (n=4 OM-MOG, n=3 vehicle). **(D)** Mean clinical scores of MOG-EAE in B6 mice injected i.d. with OM-MOG or vehicle at indicated time points (arrows), scored for the entire duration of experiment and analyzed in **(C)**. Data are from one **(A–C**, IL-10**)** or one representative of two **(C**, PD-L1; **D)** experiments. Statistical significance after multiple comparisons between groups using Student’s *t* test **(A–C)** or two way ANOVA **(D)** is shown (*p ≤ 0.05, **p ≤ 0.01, ***p ≤ 0.001).

To further understand how OM-MOG presentation by APC induces T cell anergy and given that mannan targets C-type lectin receptors expressed on myeloid cells, we next measured macrophage markers in the spleen and spinal cord of B6 EAE mice treated prophylactically or therapeutically with OM-MOG. RNA levels for *Chi3l3*, which encodes the alternative type 2 macrophage marker Ym1, were very low in spleen of mice injected with vehicle or OM-MOG (*Chi3l3*), or untreated naïve mice (*Mrc1*, *Mmp9*, *Il1β*) prior to the induction of EAE (**Figure 7A**) and increased in both injected groups during EAE with significantly increased levels in OM-MOG mice (**Figure 7A**). RNA levels for alternative macrophage markers *Mrc1* (MR, CD206), and *Mmp9*, also showed upward trends in prophylactic OM-MOG compared to vehicle mice after EAE induction, while the expression of *Il1β*, a marker of conventional pro-inflammatory (“M1”) macrophages, was equal between groups.

In experiments where OM-MOG was administered therapeutically in B6 mice with ongoing MOG-EAE, we measured RNA levels for *Chi3l3* in spleen and spinal cord, and *Il1β* in spleen, at different time points before and after OM-MOG administration. *Chi3l3* expression sharply increased in spleen and spinal cord upon EAE onset, prior to OM-MOG administration, and remained equally high in both tissues after the first injection of OM-MOG and vehicle (**Figure 7B**), consistent with alternative type 2 macrophage activation being a default response. However, *Chi3l3* expression was reduced in spleen and spinal cord after 5 injections of vehicle but maintained at high levels in spleen after 5 injections of OM-MOG, a time point when mice showed only mild residual clinical symptoms (**Figure 7D**, see *Materials and Methods*, EAE scoring). Spinal cord expression of *Chi3l3* was reduced progressively after 1 and 5 injections of OM-MOG and vehicle, possibly reflecting reduced inflammatory infiltrates in OM-MOG mice and increased inflammation in vehicle mice. Like *Chi3l3*, *Il1β* expression showed a sharp increase in spleen upon EAE onset, but thereon showed a different expression pattern to *Chi3l3*, being reduced after 1 injection, increasing again after 5 injections in both groups (**Figure 7B**).

We next investigated whether type 2 macrophage activation in the spleen by therapeutic OM-MOG was reflected by the alternative activation of APC. Increased proportions of CD11b^+^CD11c^+^ and CD11b^+^CD11c^-^ macrophages, and CD11b^-^CD11c^+^ DC producing the inhibitory costimulatory ligand PD-L1 were present in DLN and spleen of EAE mice as soon as one day after the first i.d. administration of OM-MOG compared to vehicle controls (**Figure 7C**). Also, increased proportions of CD11b^+^CD11c^+^ and CD11b^-^CD11c^+^ DC, not CD11b^+^CD11c^-^ macrophages, producing the immunosuppressive cytokine IL-10 were seen after 5 injections of OM-MOG compared to vehicle (**Figure 7C**). These results show that prophylactic and therapeutic OM-MOG enhances and maintains, respectively, a peripheral type 2 myeloid cell response during EAE that parallels its protective effects on disease.

### Human Myelin Peptides Induce Proliferation Responses in Human PBMC

**Figure 8:**
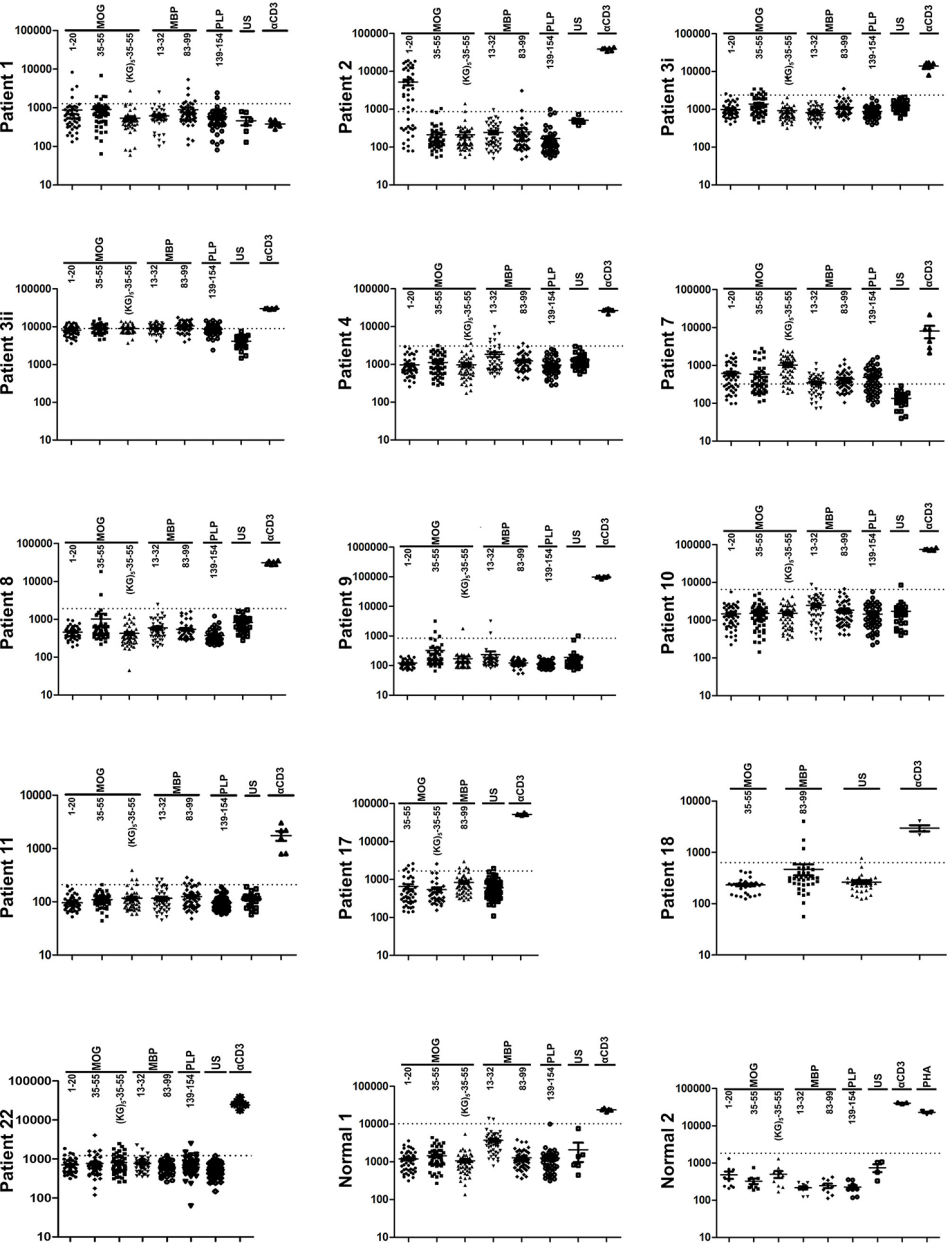
Human myelin peptides induce proliferation responses of human peripheral blood mononuclear cells (PBMC). T cell responses to myelin peptide epitopes in peripheral blood monocytes (PBMC) from patients with relapsing-remitting multiple sclerosis (RRMS) and healthy individuals (see **Table 2**). Proliferation responses were measured by thymidine incorporation (CPM) in 48 replica wells/peptide/patient. The dotted lines represent the threshold set for the mean + 3 SD of all the unstimulated wells (US), above which wells are considered positive. Peptide-specific responses were considered positive with two or more positive replicas.

Considering that OM-peptides induce antigen-specific prophylactic and therapeutic T cell tolerance across different mouse/human MHC class II in EAE models, and with view to moving studies closer to MOGAD and possibly MS, we investigated the frequencies of proliferation responses to candidate myelin autoantigens across HLA-DRB1 genotypes, in PBMC from Greek MS patients and healthy individuals (**Table 1**). Myelin-reactive T cells in MS patients and healthy individuals have been described by several investigators (37–40), but such studies in the Hellenic population are not available. The myelin peptides chosen were MBP13-32, MBP83-99, MOG1-20, hMOG35-55 (with and without the (KG)_5_ linker sequence), and PLP139-154 and responses were measured using an *in vitro* lymphocyte proliferation assay with thymidine incorporation, based on previous description (20). Positive responses were observed in 60% of MS patients screened (12/20), and neither of two healthy individuals screened (**Figure 8**; **Table 1**). The limited group of healthy individuals used here did not reveal myelin-reactive T cell proliferation responses that have been previously described by other groups in such samples, although myelin-reactive T cells in healthy individuals do differ from those in MS patients in their ability to produce inflammatory cytokines (40). In our Hellenic cohort, the incidence of proliferative responses was hMOG35-55 (9/12; patients 1,2,3,7,8,9,11,17,22) > MBP83-99 (8/12; patients 1,2,3,7,11,17,18,21) > MBP13-32 (7/12; patients 3,4,7,9,10,11,22) > MBP1-20 (6/12; patients 1,2,3,7,11,22) > PLP139-154 (4/12; patients 1,3,7,22), with many patients showing reactivity to more than one (8/12; patients 1,2,3,7,9,11,17,22), or even all five of the peptides (2/12; patients 3,7). This finding is consistent with a previous report showing a predominance of T cell responses to MOG antigens among MS patients and not healthy individuals (39). Peptide responses were not associated with particular HLA-DRB1 genotypes (**Table 1**). Taken together, these results show that myelin peptide-specific T cell responses can be readily screened and monitored in PBMC from MS patients with diverse HLA-DRB1 genotypes, as a possible selection criterion for peptide-specific immunotherapy. They also suggest that different combinations of OM-conjugated myelin antigens might be feasible for immunotherapy in patients with CNS autoimmune disease without prior need for HLA screening.

## Discussion

Here we show that an OM-conjugated myelin peptide antigen has beneficial therapeutic effects during EAE in DR2b.Ab° and B6 mice, reducing CNS inflammation and tissue damage, preserving axon integrity and increasing the expression of genes involved in myelin and neuron recovery, thereby possibly increasing repair potential in spinal cord lesions. OM-MOG rapidly increases PD-L1- and IL-10-producing myeloid cells and maintains high levels of *Chi3l3* expression in secondary lymphoid organs during EAE, and within this type 2 immune environment induces antigen-specific T cell anergy without affecting the responses of other T cells.

In the context of human disease, active EAE models closely reproduce MOGAD, a subset of inflammatory demyelinating disease originally considered as MS and distinguished by serum anti-MOG antibodies (6, 41). Demyelinating lesions in MOGAD are characterized by CD4^+^ T cell infiltrates which may react specifically with MOG or other CNS antigens (3, 42). Experimental studies show that anti-MOG antibodies alone cannot induce CNS pathology, but can induce demyelination and/or augment inflammation induced by MOG-reactive CD4^+^ T cells (4, 43). OM-peptides might therefore be effective for suppressing myelin-specific CD4^+^ T cell responses and preventing the T cell-B cell interactions that amplify inflammatory demyelination in diseases such as MOGAD. In MS, where brain lesions are dominated by clonally-expanded CD8^+^ T cells and CD20^+^ B cell infiltrates (44, 45), it is possible that OM-peptides would suppress both peptide-specific CD4^+^ and CD8^+^ T cells by bystander suppression from the alternatively-activated APC. The favorable properties of OM-conjugated MHCII-restricted myelin antigens shown in mice fully support their further investigation for safety and efficacy in clinical studies in patients with autoimmune diseases affecting the CNS.

Humanized transgenic mice expressing MHCII candidate MS susceptibility molecules HLA-DR2a (46) and HLA-DR2b (27, 47, 48) have facilitated the identification of antigenic T cell epitopes involved in CNS autoimmunity and represent an important intermediate step for testing experimental therapeutics prior to clinical trials in humans. A major goal of this study was to determine whether OM-peptides presented to T cells exclusively in the context of human MHCII molecules could be beneficial in treating EAE. DR2b.Ab° mice expressing the HLA-DR2b alleles (DRA*0101 and/or DRB1*1501), and lacking all mouse MHCII genes, support the selection of MOG- and hMOG-reactive CD4^+^ T cells that escape thymic and peripheral deletion and can induce immune responses and EAE without the need for additional human transgenes (27). Here we show that OM-MOG has beneficial prophylactic and therapeutic activities during EAE in DR2b.Ab° mice. Immunization of DR2b.Ab° mice with MOG induced the typical features of MOG-EAE, with the differentiation of Th1, not Th17, producing effector CD4^+^ T cells, and Th1 cells are sufficient to drive full-blown disease in this model. Prophylactic OM-MOG prevented the development of inflammatory demyelinating spinal cord lesions, while therapeutic OM-MOG reversed both clinical and histopathological signs of ongoing EAE in DR2b.Ab° mice, reducing spinal cord immune infiltrates, microglia responses, demyelination and axonal damage. To bypass difficulties in breeding and maintenance of DR2b.Ab° mice, supporting studies carried out in B6 mice showed that OM-MOG treatment preserved axons in spinal cord lesions without measurable changes in remyelination and increased spinal cord RNA levels of *Mbp* and *Snap25* above pretreatment levels measured at disease onset (clinical score 2). These results may have several wider implications for the understanding of pathology and immunotherapy in CNS demyelinating diseases: 1) axon pathology is associated with immune cell infiltration of CNS tissue; 2) spontaneous remyelination proceeds independently of an autoimmune directed response, at least at the time point studied (day 25 post-immunization for EAE); 3) immunotherapies that inhibit autoimmune responses to CNS myelin (e.g. OM-peptides), preserve axons and thereby might increase the repair potential of CNS tissues.

The mechanism by which mannosylated and mannan-conjugated myelin antigens induce immune tolerance in EAE has been elusive. Mannose motifs are common in pathogens and their uptake is mediated by C-type lectin receptors such as the mannose receptor (MR; CD206), that are expressed at high levels by immature myeloid cells, leading to presentation by MHC class I and II to CD8^+^ and CD4^+^ T cells, respectively (49). Early studies using mannan conjugated to a peptide of the MUC1 tumor protein described strong type 1 and 2 immune responses and protection against MUC1-expressing tumors in mice (50, 51), and humans (52). However, in an autoimmune environment such as EAE, mannosylated and mannan-conjugated peptides clearly induce antigen-specific immune tolerance (23, 24), and this is further demonstrated here where prophylactic OM-MOG did not prevent the induction of EAE by a different myelin antigen, PLP178-191 in B6 mice, or reduce *ex vivo* T cell proliferation responses to polyclonal stimulation with anti-CD3 antibody. The tolerance mechanism is therefore restricted by interactions between MOG-presenting APC and MOG-specific T cells. Similar effects were reported for mannosylated PLP139-151, which protects against PLP139-151-induced EAE in SJL mice, and enhances the expansion of T cells with reduced peptide-specific proliferation but equal expression and secretion of IFN-γ, IL-10, and IL-4 in SJL mice (23). Here, we recorded several features of anergy, an acquired state of T cell functional hyporesponsiveness (53), in MOG-specific T cells exposed to OM-MOG *in vitro* and *in vivo*; loss of the ability to produce and respond to the autocrine growth factor IL-2; reduced proliferation in response to MOG; high production of FR4 by CD4^+^FoxP3^-^CD44^+^ cells in the spleen of therapeutic OM-MOG B6 mice. High expression of FR4 and CD73 are shared by anergic CD4^+^FoxP3^-^ T cells and CD4^+^Foxp3^+^ Treg and both molecules are thought to be functionally involved in suppressing immune responses to self-antigens during pregnancy and in autoimmune disease models, in part by allowing the generation of Treg (36). *In vivo*, anergy is induced and maintained by chronic interaction with self-peptide-MHC class II complexes in the absence of pro-inflammatory co-stimulation signals, and it is possible that OM-MOG reproduces this effect.

MR is highly expressed by alternatively activated type 2 macrophages, which are essential for limiting inflammation, scavenging debris and tissue remodeling (54). Here, prophylactic and therapeutic OM-MOG maintained or induced the alternative activation of myeloid cells even in the presence of strong pro-inflammatory stimuli provided by immune adjuvants and the EAE environment. The expression of *Chi3l3*, which encodes Ym1, is a marker for type 2 macrophages and was increased in the spleen and spinal cord at the onset of EAE, and in spleen was further increased by prophylactic OM-MOG and maintained by therapeutic OM-MOG. Recently discovered beneficial functions of mouse Ym1 are relevant for MS, because high levels of human chitinase-like molecules are expressed in the brain during acute demyelination (55). Ym1-expressing CD11b^+^ microglia are located in the stem cell niche of subventricular zone in the adult mouse brain, and in inflammatory brain lesions in EAE, and Ym1 promotes oligodendrogenesis (56). In a colitis model, Ym1^+^Ly6C^hi^ monocytes expand in the bone marrow during the recovery phase, produce high levels of IL-10 and contribute to the resolution of inflammation (57). Here, therapeutic tolerance by OM-MOG was also associated with significantly increased IL-10 production by CD11c^+^CD11b^+^ and CD11c^+^CD11b^-^ DC in the spleen. A role for MR engagement in this effect is supported by a previous report that specific engagement of MR on immature monocyte-derived DC with an agonist anti-MR antibody induced maturation of DC with an anti-inflammatory immunosuppressive profile, including production of IL-10, and ability to induce anergy and regulatory properties in T cells (58). Importantly, a single i.d. injection of OM-MOG was sufficient to rapidly (within 24 h) upregulate production of PD-L1 by CD11b^+^ macrophages and CD11c^+^ DC in both DLN and spleen of mice with active EAE. Both PD-L1 and PD-L2 provide strong co-inhibitory signals *via* PD-1 on T cells during T cell activation that result in T cell anergy (59).

Together our results show two main beneficial effects that underlie the mechanism of immune tolerance by OM-peptides. First is rapid deviation of the peripheral immune response to a type 2 profile, with myeloid cells expressing high levels of PD-L1 and IL-10, and secondary lymphoid organs upregulating Chi3l3 expression. Second is anergy induction in antigen-specific T cells. We hypothesize that OM binding to the mannose receptor on myeloid cells induces generalized type 2 immune deviation, while the simultaneous presentation of antigen to T cells by the OM-antigen-activated APC induces anergy specifically in the antigen-specific T cells but not T cells of other specificities, thereby resulting in antigen-specific T cell tolerance. This hypothesis remains to be tested by comparing populations of CD4^+^ T cells with different antigen specificities isolated from the OM-peptide tolerized mice.

Considering that the polymorphic nature of the human MHC presents a challenge for the translation of antigen-specific targeting strategies into humans, we also investigated whether Greek patients diagnosed with MS with diverse HLA-DRB1 genotypes can be screened for their T cell responses to disease-associated myelin peptides for the purposes of patient selection and stratification in future clinical trials. The five peptides chosen, MBP13-32, MBP83-99, MOG1-20, hMOG, and PLP139-154, are all targets of the high avidity autoimmune T cell response in MS (20). We show here that MS patients can be readily screened for T cell responses using peripheral blood cells in a simple *in vitro* lymphocyte proliferation assay. Notably, high proportions of MS patients in our Hellenic cohort showed responses to hMOG along with other disease-associated myelin peptides independent of HLA-DRB1 genotype. Also, OM-peptides were found to induce tolerance across diverse mouse/human MHC class II barriers including DR2b. Taking this information into account, it is possible that different combinations of OM-conjugated myelin peptides might be useful for inducing peripheral antigen-specific T cell tolerance in human autoimmune CNS diseases in which autoantigens are defined, such as MOGAD (3), without prior need for HLA selection.

## Data Availability Statement

The raw data supporting the conclusions of this manuscript will be made available by the authors, without undue reservation, to any qualified researcher.

## Ethics Statement

The studies involving human participants were reviewed and approved by the Scientific Committee of Aeginition Hospital, National and Kapodistrian University of Athens, Greece. Licence 283/13‐05‐2015 ΑΔΑ: 7Β∑Η46Ψ8Ν2‐Β66. The patients/participants provided their written informed consent to participate in this study. Written informed consent was obtained from the human individual(s) for the publication of any potentially identifiable images or data included in this article. The animal study was reviewed and approved by Committee for Evaluation of Experimental Procedures, Department of Experimental Animal Models, Hellenic Pasteur Institute (Presided by Dr P Andriopoulos pandriopoulos@patt.gov.grfor the Hellenic Republic, General Secretariat for Agricultural Economy, Veterinary and Licenses). Licence number 2580/31-05-2018.

## Authors Contributions

AD designed and performed prophylactic and treatment EAE experiments, neuropathological, proliferation and FACS analyses, and analyzed results; MAv. designed and performed the DC-T cell assays, human PBMC proliferation assays, FACS analyses, analyzed results, and maintained the DR2b.Ab° mouse colony; ME designed and performed human PBMC and mouse lymphocyte response assays, FACS analyses, EAE and priming experiments, and analyzed results; IP performed quantitative RT-PCR analyses, and analyzed results; IK established the DR2b.Ab° mouse colony and helped set-up the EAE protocol, performed EAE experiments, and analyzed results; VT set-up the EAE protocol in DR2b.Ab° mice and the DC-T cell assay protocol, performed EAE and priming experiments and analyzed results; FL performed EAE and priming experiments; TT designed, synthesized, purified and characterized myelin peptide analogues; LTJ generated DR2b.Ab mice, and set-up protocols for mouse genotyping; WM and TR performed electron microscopy; M-EA synthesized, purified and characterized myelin peptide analogues; JM designed myelin peptide analogues; MAn performed HLA genotyping, provided blood from MS patients, and supervised Hellenic MS patient cohort; HL performed, analyzed and interpreted neuropathology; LP was responsible for overall experimental design and management of project, analyzed and interpreted data, wrote paper. All authors edited the final version of the paper. All authors contributed to the article and approved the submitted version.

## Funding

This research was co‐financed by European Union and Greek national funds by The Management and Implementation Authority for Research, Technological Development and Innovation Actions (MIA-RTDI/ ΕΥΔΕ-ΕΤΑΚ) of the Hellenic Ministry of Development and Investments, through the Operational Program Competitiveness, Entrepreneurship and Innovation, under the call RESEARCH – CREATE – INNOVATE (project code T1EDK-01859).

## Conflict of Interest

MA was employed by the company Vianex. S.A.

The remaining authors declare that the research was conducted in the absence of any commercial or financial relationships that could be construed as a potential conflict of interest.

## Supplementary Material

Supplementary Figure 1 Therapeutic OM-MOG preserves axons in spinal cord lesions during MOG-EAE in B6 mice. **(A)** Numbers of non-collapsed myelinated axons/mm^2^ and **(B)** collapsed axons/mm^2^ in groups of mice shown in **Figure 3 (F–H)**. Data and statistical significance are derived from one experiment after pairwise comparisons between samples from different mouse groups using Kruskal-Wallis test (**p* ≤ 0.05, ****p* ≤ 0.001).

Supplementary Figure 2 OM-MOG induces semi-maturation of DR2b.Ab° DC. **(A, B)** FACS of bone marrow-derived CD11c^+^ DC from DR2b.Ab° mice, loaded *in vitro* with peptides or incubated bone marrow-derived with LPS, for cell surface expression of maturation markers CD40 **(A)** and CD80 **(B)**. Data are from one representative of three independent experiments. Statistical significance after multiple comparisons between groups using one-way ANOVA is shown (**p*≤ 0.05, ***p* ≤ 0.01, ****p* ≤ 0.001).

## Abbreviations

APP, amyloid precursor protein; APC, antigen presenting cells; B6, C57/BL6 mice; *Chi3l3*, mouse chitinase-like 3 gene; CFA, complete Freund’s adjuvant; CFSE, carboxyfluorescein succinimidyl ester; DC, dendritic cells; DLN, draining lymph nodes; dpi, days post immunization; DR2b.Ab°, transgenic mice expressing *DRA*0101* and *DRB*1501* and lacking all murine MHCII genes; EAE, experimental autoimmune encephalomyelitis; FR4, folate receptor 4; *Gusb*, glucoronidase beta gene; *H2-Ab1*, mouse H-2 class II histocompatibility antigen, A beta chain gene; HLA, human leukocyte antigen; hMOG35-55, human myelin oligodendrocyte glycoprotein 35-55 peptide; Iba1, ionized calcium-binding adapter molecule 1; LFB, luxol fast blue; MBP, myelin basic protein; MFI, mean fluorescence intensity; MHCII, major histocompatibility complex class II; MOG, murine myelin oligodendrocyte glycoprotein; MOGAD, MOG antibody associated disorders; *Mmp9*, mouse matrix metalloproteinase 9 gene; MR, mannose receptor; *Mrc1*, mouse mannose receptor c-type 1 gene; MS, multiple sclerosis; MUC1, mucin 1; OM, oxidized mannan; PD-L1, programmed death-ligand 1; PFA, paraformaldehyde solution in PBS; PLP, proteolipid protein; PTx, *Bordetella* *pertussis* toxin; RT, room temperature; SI, stimulation index; *Snap25*, mouse synaptosomal-associated protein 25kDa gene; TCR, T cell receptor, Th, CD4^+^ T helper cells; Th1, interferon-γ- (IFN-γ) producing Th cells; Th17, IL-17-producing Th cells; Treg, regulatory T cells.
