# Supplementary figures and images for: Mannan-MOG35-55 Reverses Experimental Autoimmune Encephalomyelitis, Inducing a Peripheral Type 2 Myeloid Response, Reducing CNS Inflammation, and Preserving Axons in Spinal Cord Lesions
Source: Front Immunol. 2020 Nov 19;11:575451. doi: 10.3389/fimmu.2020.575451 (PMC7711156; doi:10.3389/fimmu.2020.575451)

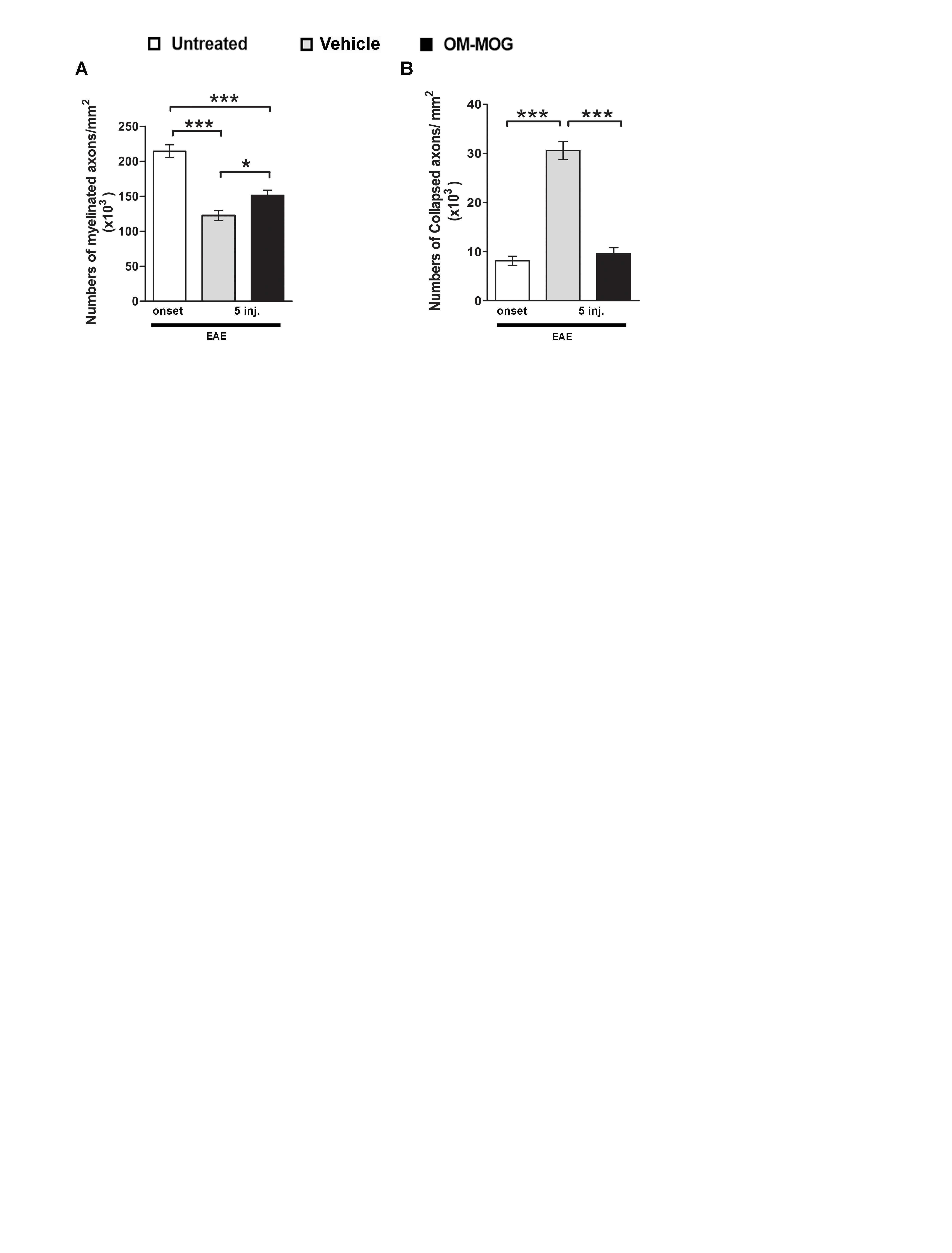

Supplement: Supplementary Figure 1 — Therapeutic OM-MOG preserves axons in spinal cord lesions during MOG-EAE in B6 mice. (A) Numbers of non-collapsed myelinated axons/mm2 and (B) collapsed axons/mm2 in groups of mice shown in Figure 3 (F–H) . Data and statistical significance are derived from one experiment after pairwise comparisons between samples from different mouse groups using Kruskal-Wallis test (*p ≤ 0.05, ***p ≤ 0.001). [file Image_1.jpeg]

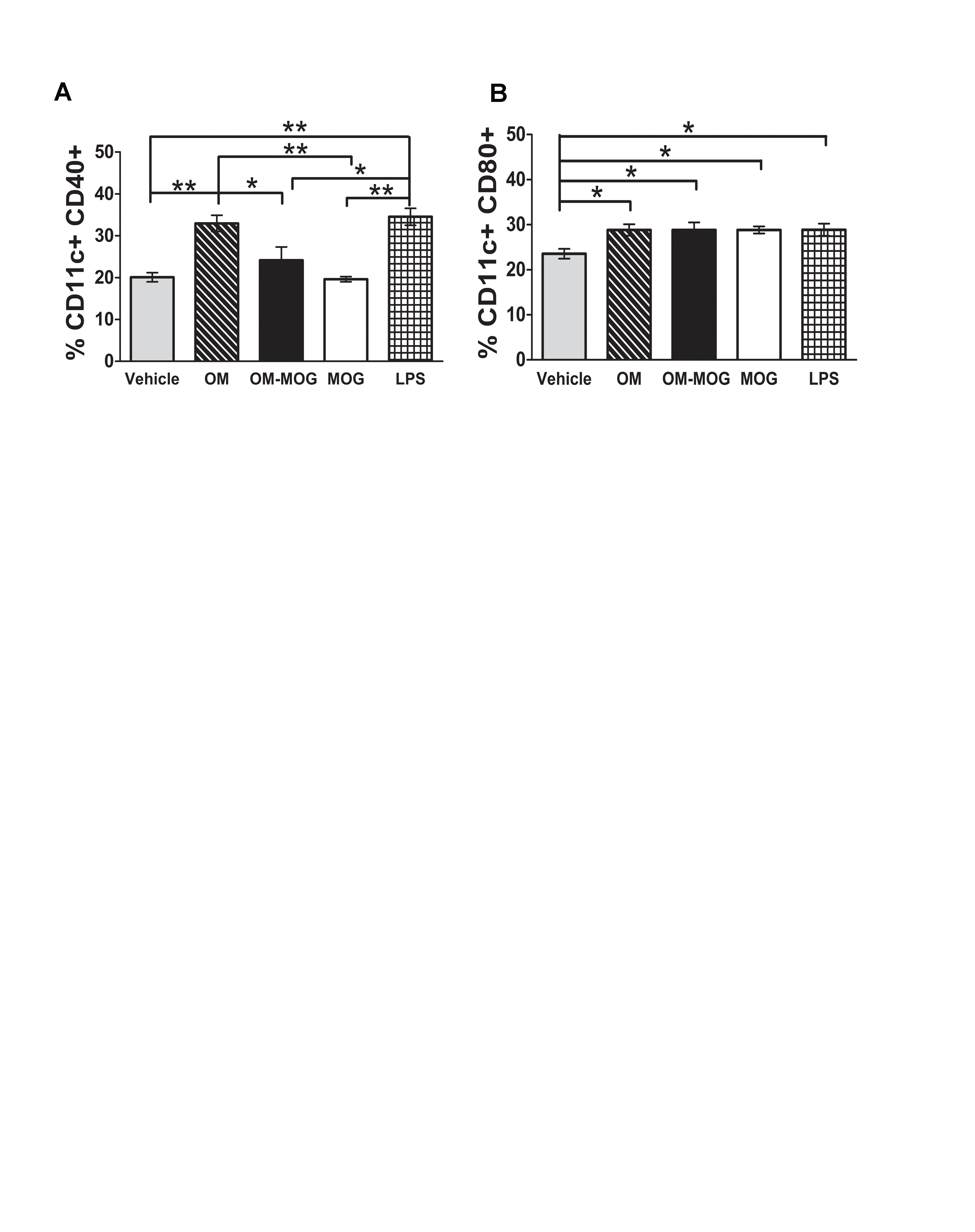

Supplement: Supplementary Figure 2 — OM-MOG induces semi-maturation of DR2b.Ab° DC. (A, B) FACS of bone marrow-derived CD11c+ DC from DR2b.Ab° mice, loaded in vitro with peptides or incubated bone marrow-derived with LPS, for cell surface expression of maturation markers CD40 (A) and CD80 (B). Data are from one representative of three independent experiments. Statistical significance after multiple comparisons between groups using one-way ANOVA is shown (*p≤ 0.05, **p ≤ 0.01, ***p ≤ 0.001). [file Image_2.jpeg]
