# Supplementary material for: Mannan-MOG35-55 Reverses Experimental Autoimmune Encephalomyelitis, Inducing a Peripheral Type 2 Myeloid Response, Reducing CNS Inflammation, and Preserving Axons in Spinal Cord Lesions
Source: Front Immunol. 2020 Nov 19;11:575451. doi: 10.3389/fimmu.2020.575451 (PMC7711156; doi:10.3389/fimmu.2020.575451)
Supplement: Supplementary file 3 [file Presentation_1.pdf]

Autoimmune demyelinating disorders of the central nervous system (CNS) are a major cause of neurologic disability in young adults. In multiple sclerosis the autoimmune targets of adaptive T lymphocyte and autoantibody responses are not yet fully understood, but in other diseases such as myelin oligodendrocyte glycoprotein (MOG)-antibody associated inflammatory demyelinating disease, the myelin protein MOG is strongly implicated as a target of CNS-infiltrating CD4+ T cells. Currently available immunomodulatory drugs are rather non-specific and impair host defense responses. The development of autoantigen-specific immunotherapies that would selectively inhibit the immune cells that attack and destroy CNS myelin is a major goal for improving treatment. Here we show that intradermal injection of MOG antigen conjugated to an oxidized form of the yeast polysaccharide mannan induces a protective “type 2” innate immune environment in mice that specifically inhibits the CD4$^+$ T autoimmune attack on myelin and rapidly reverses the neurological deficits associated with autoimmune demyelination. In the spinal cord, mannan-MOG treatment has anti-inflammatory, neuroprotective and tissue repair activities, and treatment is effective in both wildtype and humanized HLA-DR2 mice. The results suggest that patients affected by CNS demyelinating diseases with known autoimmune targets might be candidates for peptide-specific therapy with mannan-conjugated autoantigens.
